# A Simple, Lightweight, and Low-Cost Customizable Multielectrode Array for Local Field Potential Recordings 

**DOI:** 10.1523/ENEURO.0212-23.2023

**Published:** 2023-08-28

**Authors:** Richard Quansah Amissah, Abdalla M. Albeely, Elise M. Bragg, Melissa L. Perreault, Wilder T. Doucette, Jibran Y. Khokhar

**Affiliations:** 1 Department of Biomedical Sciences, Ontario Veterinary College, University of Guelph, Guelph, Ontario N1G 2W1, Canada; 2 Department of Anatomy and Cell Biology, Schulich School of Medicine and Dentistry, Western University, London, Ontario N6A 5C1, Canada; 3 Department of Psychiatry, Dartmouth Hitchcock Medical Center, Lebanon, New Hampshire 03756; 4 Geisel School of Medicine at Dartmouth, Hanover, New Hampshire 03755-1404

**Keywords:** electrodes, electrophysiology, *in vivo*, local field potential, multielectrode array

## Abstract

Local field potential (LFP) recording is a valuable method for assessing brain systems communication. Multiple methods have been developed to collect LFP data to study the rhythmic activity of the brain. These methods range from the use of single or bundled metal electrodes to electrode arrays that can target multiple brain regions. Although these electrodes are efficient in collecting LFP activity, they can be expensive, difficult to build, and less adaptable to different applications, which may include targeting multiple brain regions simultaneously. Here, the building process for a 16-channel customizable multielectrode array (CMEA) that can be used to collect LFP data from different brain regions simultaneously in rats is described. These CMEA electrode arrays are lightweight (<1 g), take little time to build (<1 h), and are affordable ($15 Canadian). The CMEA can also be modified to record single-unit and multiunit activity in addition to LFP activity using both wired and wireless neural data acquisition systems. Moreover, these CMEAs can be used to explore neural activity (LFP and single-unit/multiunit activity) in preliminary studies, before purchasing more expensive electrodes for targeted studies. Together, these characteristics make the described CMEA a competitive alternative to the commercially available multielectrode arrays for its simplicity, low cost, and efficiency in collecting LFP data in freely behaving animals.

## Significance Statement

Dysregulated neural oscillatory activity is present in multiple neuropsychiatric and neurodegenerative disorders. To accurately diagnose and treat these disorders, it is important to understand the mechanisms that underlie neural dysfunction. Several types of neural pathologies have been studied including LFP activities, which represent a summation of population-level neuronal activity recorded from within the brain. Although several commercially available electrodes for studying LFP activity exist, they are expensive, not easily adaptable, and difficult to build. Here, we describe a simple CMEA that is lightweight, easy to build, affordable, and adaptable to a number of applications.

## Introduction

The brain is a complex organ made up of neuronal and non-neuronal cells (Freire-Regatillo et al., 2017) that work together to control processes including cognition and learning. Most neurons communicate by releasing neurotransmitters (Kaeser and Regehr, 2014), leading to membrane depolarization/hyperpolarization (Nicoll, 1988) and the generation of action potentials (APs; Palay et al., 1968; Barnett and Larkman, 2007). Neural activity can be measured *in vitro* and *in vivo*. *In vitro*, APs can be studied in brain slices using tools such as glass micropipettes (Wickenden, 2014). Although this technique is useful for understanding AP mechanisms and cellular membrane properties, it is not ideal for studying the relationship between neural activity and animal behavior. To overcome this challenge, *in vivo* electrophysiological recordings, which can be performed through noninvasive and invasive (involving surgery) techniques, can be used. An example of an *in vivo* noninvasive technique is electroencephalography (EEG), one of the oldest techniques for investigating neural function (Nunez and Srinivasan, 2006; Schomer and Da Silva, 2011), which is used to study sleep disorders, epilepsy, and coma in humans (Im and Seo, 2016). However, because of the difficulty in locating the source of the EEG signal and the firing patterns of the neurons (Nunez and Srinivasan, 2006), researchers turn to invasive *in vivo* techniques, mostly used in animal models. One example of an invasive *in vivo* technique is fiber photometry, which relies on the expression of genetically encoded calcium indicators that provides insight into neuronal activity by measuring changes in calcium levels (Legaria et al., 2022). The technique offers complementary advantages over EEG, in that it enables cell-type-specific targeting and monitoring of neuronal activity and has better spatial resolution compared with EEG. Other invasive *in vivo* techniques involve the implantation of metal electrodes, which have low high-frequency impedance, high signal-to-noise ratio, and good mechanical properties (Im and Seo, 2016), in the brain to collect APs from neurons (O’Keefe and Dostrovsky, 1971; Ranck, 1973). APs identified to be produced by single neurons are called single units, whereas those from multiple neurons are multiunits (Wickenden, 2014). Implanted electrodes can also collect local field potential (LFP) activity, which are synaptic currents generated through the summed synchronous synaptic activity of neurons (Dzirasa et al., 2011; Harris et al., 2017; Haumesser et al., 2017). Although EEG, calcium, and LFP activity represent neural population activity, LFPs have a better spatial and temporal resolution, are stable and robust across changes in the electrode sensitivity (Im and Seo, 2016; Zhang et al., 2022), and are less susceptible to electrode micromotion effects (Martini et al., 2020). Some benefits of LFP recordings over photometry include the ease of not requiring viral injections and delays related to expression, as well as the potential to target a much greater number of sites. Moreover, LFPs can be used to evaluate field–field coherence, a measure of the correlation between the activity of two brain regions in a specific frequency band (Fries, 2005). This can provide insights into the functional connectivity between different brain regions and how this changes over time (Aoi et al., 2015; Schneider et al., 2021). Several metal electrodes, including multielectrode arrays (MEAs), which enable neural activity to be recorded from multiple sites simultaneously (Im and Seo, 2016; Kim et al., 2018; Zhang et al., 2022), are available commercially. However, they are expensive, fragile, and ideal for studying neural activity either in one region at a time or in a few, adjacent, cortical regions (Mohapatra et al., 2022), which limits their applicability for studying entire neural circuits. Therefore, here, we propose a simple, lightweight, and affordable customizable MEA (CMEA) that can be used to record LFP activity from multiple brain regions simultaneously (Dwiel et al., 2019; Nelong et al., 2019; Albeely et al., 2022; Henricks et al., 2022; Jenkins et al., 2022; Albeely et al., 2023; Quansah Amissah et al., 2023) and can also be used for stimulating brain regions to study rodent models of focal brain stimulation (Doucette et al., 2015; Dwiel et al., 2019).

## Materials and Methods

All animal procedures were performed in accordance with the regulations of the Institutional Animal Care Committee and those set out by the Canadian Council on Animal Care.

**Figure 1.**
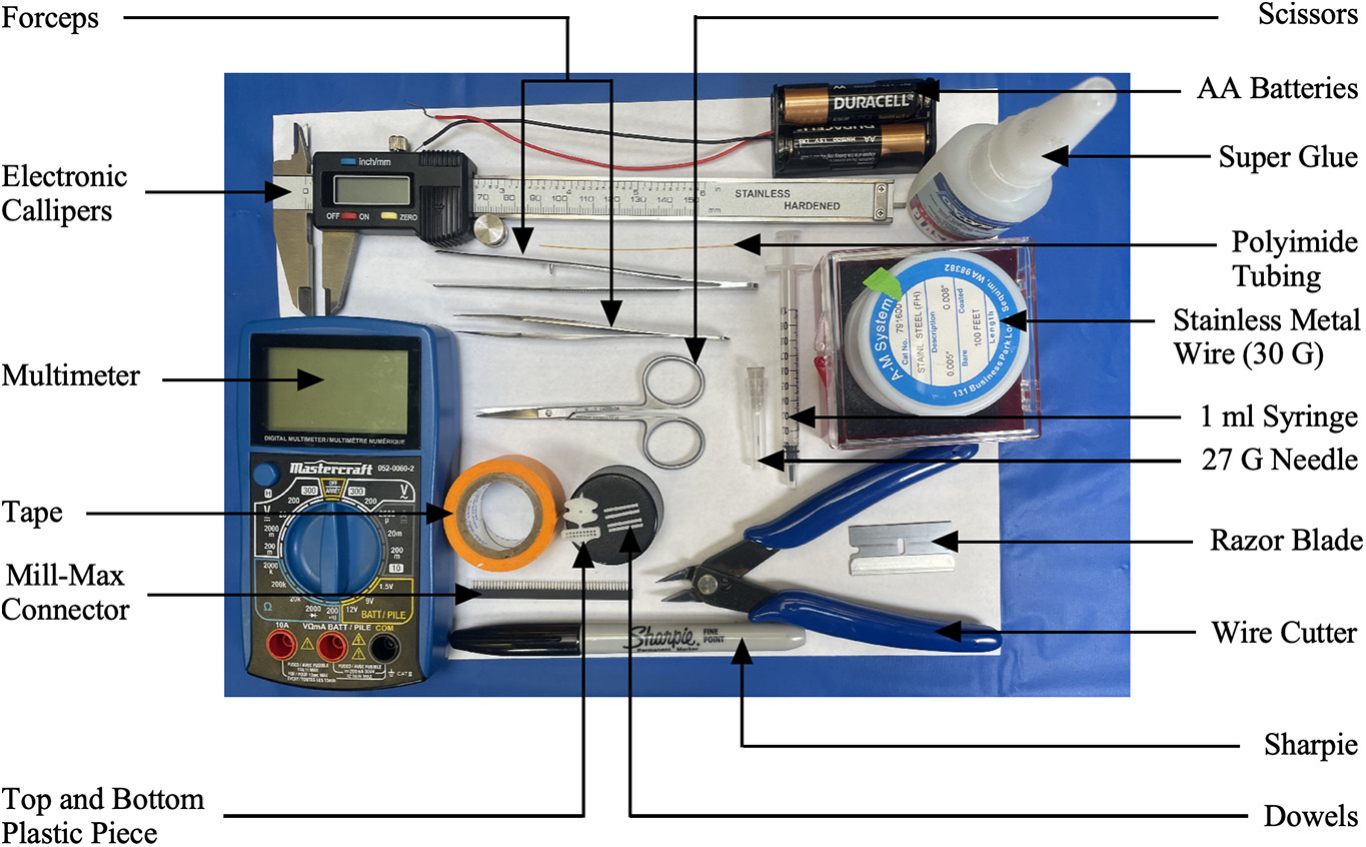
The materials required to build the multielectrode array.

Here, a unipolar CMEA was constructed to target different brain regions simultaneously with coordinates for these regions obtained from the Paxinos rat brain atlas (Paxinos and Watson, 2013). The items required to build the multielectrode array and their cost are provided in Figure 1 and Table 1 (Extended Data Table 1-1, alternative supplier list).

**Table 1:** Bill for materials for the components of the complete customized multielectrode array

Item	Product number	Vendor	Quantity	Cost per CMEA
Delrin plastic pieces		Amazon	1 (top and bottom pieces)	3.33
Plastic dowel	2500470500	DigiKey	1	0.22
Polyimid tubing	95820-02	VWR International	1 (<35 mm)	1.82
PFA-coated stainless steel wire	791600	A-M Systems	1 (18 inches)	2.93
Mill-Max connector	853–93-100-10-001000	DigiKey	1 (2 × 9 pins)	4.04
Superglue	855977000078	Amazon	1 (100 µl)	0.05
Male/male zero insertion force Zif-Clip to Mill-Max adaptor	8501005010001000	DigiKey	1 (2 × 9 pins)	2.5
Total cost				$15.91 Canadian

10.1523/ENEURO.0212-23.2023.t1-1 Table 1-1 Alternative supplier list. Download Table 1-1, DOCX.

### Building the multielectrode array

**Figure 2.**
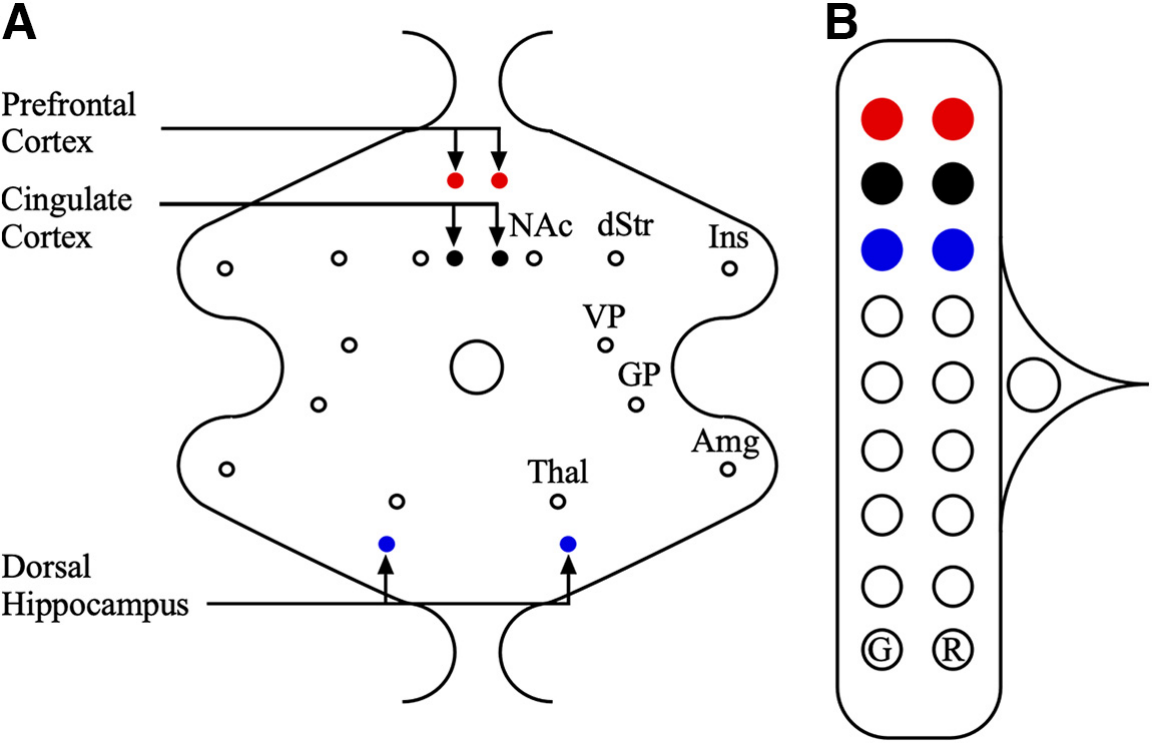
Schematic showing bottom and top plastic pieces of the multielectrode array. ***A***, Schematic showing the bottom plastic piece with cannula holes indicating coordinates for different brain regions. Three brain regions are targeted here—the prefrontal cortex (red dots), cingulate cortex (black dots), and dorsal hippocampus (blue dots). ***B***, Schematic showing the top plastic piece with color-coded pinholes corresponding to electrodes from the bottom plastic piece. G, Pinhole for the ground electrode; R, pinhole for the reference electrode; Amg, amygdala; dStr, dorsal striatum; GP, globus pallidus; Ins, insula; NAc, nucleus accumbens; thal, thalamus; VP, ventral pallidum.

Before building the CMEA, a map or schematic of the electrode design should be made with all information needed for building (Fig. 2). This should include brain regions being targeted, the anteroposterior, mediolateral, and dorsoventral coordinates for the targeted brain regions, the lengths of each cannula needed, the gauge of the cannula, the gauge of the wire, the length wires will be trimmed to, and if gold pins are needed. The map should show the layout of both top and bottom plastic pieces, where each cannula will be placed, and where each wire will be pinned.

**Figure 3.**
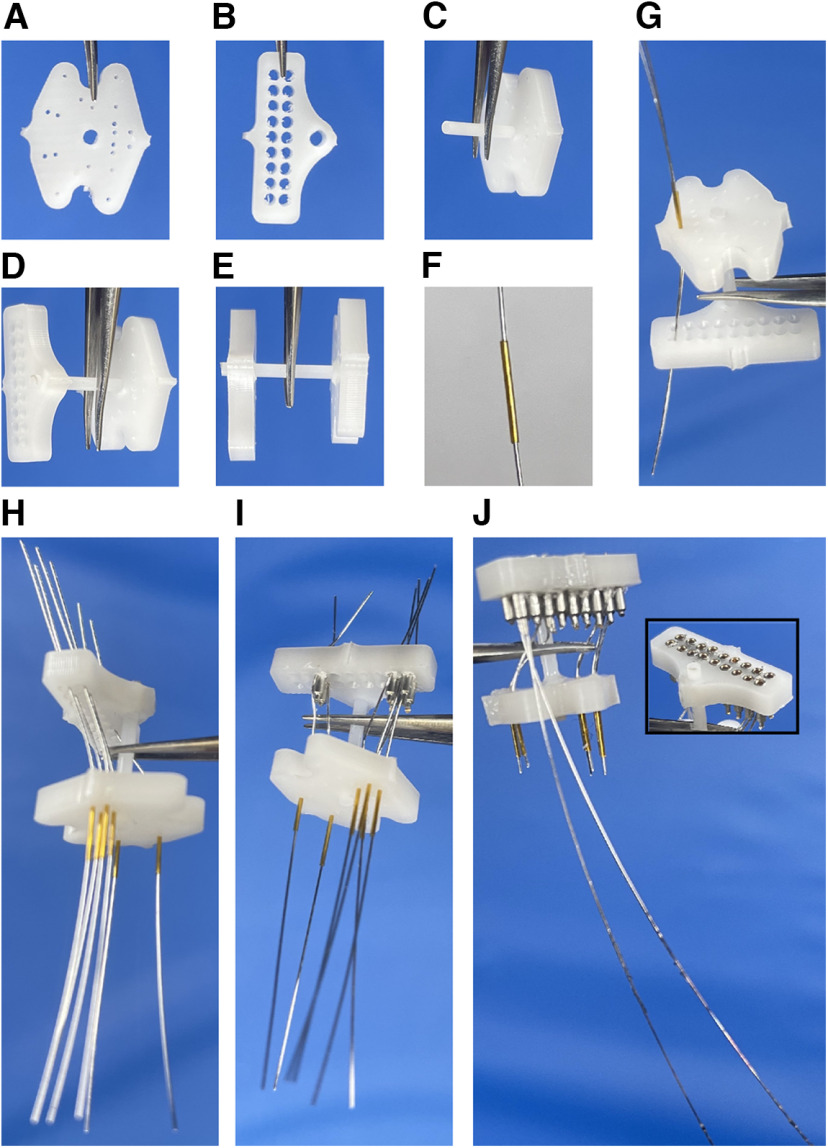
Steps for building the multielectrode array. ***A–J***, Bottom (***A***) and top (***B***) plastic pieces were joined using a dowel (***C***, ***D***, ***E***). A cannula was cut to the appropriate length, and a precut wire was inserted through (***F***). All cannulas with precut wires were threaded through the corresponding holes in the top and bottom plastic pieces (***G, H***). Pins were inserted into the holes in the top plastic piece to stabilize the wires and to create a connection between the wires and the pins (the insulation is sheared by the pins to allow connection; ***I***). After insertion of all pins (***J***), extra wires protruding from the top plastic piece were cut, and those from the cannulas were shortened to a predetermined length (according to the desired DV coordinates). The reference and ground wires were left intact. Inset (***J***), The top of the top plastic piece on completion of the multielectrode array.

Based on the schematic (Fig. 2), which contains the brain regions of interest and their coordinates (anterior–posterior and medial–lateral), the models for the top and bottom plastic pieces were created using computer-aided design (CAD) software [two sample wire frame Initial Graphics Exchange Specification (IGES) files have been included; https://osf.io/ZXF5T/; Extended Data 1] that can be modified for the brain regions of interest). The sizes of the top and bottom plastic pieces were chosen according to the average size of the head of the rat. The model was then converted into G-CODE, using computer-aided manufacturing, and uploaded into a computer numerically controlled (CNC) machine to carve out the templates for the top and bottom plastic pieces from the Delrin plates (150 × 150 × 6 mm; DuPont) according to the dimensions specified in the CAD file. 

10.1523/ENEURO.0212-23.2023.ext1 Extended Data 1 Sample wireframe IGES files for the Delrin templates (see DOI 10.17605/OSF.IO/ZXF5T). Download Extended Data 1, ZIP file.

### Steps for building the multielectrode array

1. Select one bottom and one top plastic piece (Fig. 3*A*,*B*).
  - Check each piece to be sure the cannula holes and the pinholes are well centered and not too close to the edges, which might make them more likely to break.
2. Enlarge the cannula holes if necessary.
  - Using a needle (27 or 30 gauge), push with a twisting motion until the needle goes all the way through the existing holes.
3. Insert a plastic dowel into the larger/center hole of the bottom plastic piece (Fig. 3*C*).
  - Be sure that the end of the dowel is flush with the flattest side of the bottom plastic piece (which will now be the bottom side).
  - You may need to enlarge the beginning of the hole slightly with a smaller-gauge needle to help start the dowel.
4. Slide the top plastic piece onto the plastic dowel (Fig. 3*D*,*E*).
5. Cut the cannula to the appropriate length.
  - For the most efficient cutting, measure and mark each length needed on a piece of tape on the lab bench. Lay the cannula along the markings and cut with a new razor blade. Check the cut lengths with calipers.
  - The cannula length can be determined by identifying the dorsoventral coordinate of the brain region of interest (*x*), the thickness of the bottom plastic piece (*y*), and how far the metal electrode (wire) will extend outside the cannula (*z*). The length of the cannula was therefore calculated using the formula: *x* + *y* – *z*.
6. Cut the wire from the spool into 2 inch lengths.
7. Thread a single wire through each precut cannula (Fig. 3*F*). Repeat for all cannulas.
8. Insert the correct length cannula with wire into the corresponding holes in the top and bottom plastic pieces (Fig. 3*G*). Repeat until all wires and cannulas are in the corresponding holes in the top and bottom plastic pieces (Fig. 3*H*).
  - Insert the cannula with wire into the underside of the bottom plastic piece and be sure the top of the cannula is flush with the top side of the bottom plastic piece (opposite of the plastic dowel).
  - Insert the wire in each cannula into the corresponding hole in the top plastic piece.
    - Using forceps and a magnifying glass make threading the wire easier.
    - Use the electrode map/schematic (Fig. 2) to confirm where each wire should be threaded.
9. Insert a female pin, previously removed from a Mill-Max connector strip, into the pinhole from the top of the top plastic piece (Fig. 3*I*).
  - Insert the pin with the wire toward the outside of the top plastic piece. This ensures the wire is stabilized against the thickest wall and will make the best connection with the pin.
  - Be sure the pin holds the wire in place in the pinhole. If the wire is very fine, and the pin goes fully into the pinhole without any resistance, it may not shear the protective coating on the wire and therefore not make a connection with the pin. A gold female pin may need to be used instead to make a good connection.
10. Repeat with each cannula until all wires are threaded and pinned.
  - Some designs (tetrodes or single-wire designs to access multiple regions with similar AP and ML coordinates but different DV coordinates) may call for multiple wires to be threaded through one cannula. In this case, the wires can be bundled and held together with dextrose and subsequently threaded through the cannula and the top plastic piece.
  - The electrode wires could be threaded for either unipolar or bipolar recording. Thread one wire (ground wire) through the designated hole on the top plastic piece and insert a pin to hold it in place. Be sure to deinsulate the free end of the ground and reference wires using a lighter.
11. Insert pins into any remaining empty pinholes in the top plastic piece (Fig. 3*J*).
12. Push the top and the bottom plastic pieces closer together once all wires have been threaded and pined (Fig. 3*J*).
  - Pull wires taut from the nonpinned ends to decrease the space between the top and bottom pieces.
13. Evaluate the connection between each pin and wire using the bubble test.
  - Be sure both wires are firmly connected to a 9 V battery. Fill one well on a plastic well plate with saline and insert the end of one wire attached to the 9 V battery into the saline. Hold the wire ends of the electrode in the saline and, one by one, touch the end of the other wire connected to the battery to the top of each pin of the electrode. Watch for bubbles on or coming from the tip of the electrode wire that corresponds to the pin being touched.
  - If no bubbles (or very few) form, the connection between the wire and pin may be weak.
  - Look for bubbles coming from wires not being touched as this may indicate cross-talk between pins or wires.
  - A magnifying glass or microscope may be helpful.
14. Fix weak connections or nonconnections and retest.
  - After identifying the weak connections or nonconnections, gently push each pin out from the bottom of the top plastic piece.
  - Pull out the electrode from the corresponding hole.
  - Cut a new electrode of the same length and thread it through the cannula corresponding to the pinhole from which the pin was pushed.
  - Insert the cannula with the electrode into the corresponding hole in the bottom plastic piece, ensuring that the cannula is flush with the top part of the bottom plastic piece.
  - Guide the electrode through the bottom part of the top plastic piece into the corresponding pinhole.
  - Afterward, push the pin into the pinhole in the top plastic piece as done previously.
  - Retest until all pins have good/strong connections with corresponding wires and ensure there is no cross-talk between neighboring pins and/or wires.
15. Carefully apply superglue to the bottom of the cannula and the underside of the bottom plastic piece to hold it in place.
  - Too much glue can pull/shift the cannula as it dries, but too little will not hold the cannula in place.
  - Avoid getting glue in the ends of the cannula to prevent clogging.
16. Apply superglue to all movable parts using a 1 ml syringe and a 27 gauge needle.
  - Apply superglue to the dowel around the top plastic piece, the bottoms of the pins, and the wires as they enter the cannula at the top of the bottom plastic piece.
  - Be careful not to get glue inside the pins on the top plastic piece.
  - Hold the plastic pieces in place as the superglue dries.
17. Remove the ends of the wires protruding from around the pins on the top of the customized multielectrode array (Fig. 3*J*, inset).
  - Use a magnifying glass, a pair of scissors, and forceps.
  - Grasp the end of the wire with forceps and gently pull it taut.
  - Afterward, make a circular motion with the electrode wire while keeping the gentle tension. This will create a pressure point in the wire where it is pinched between the plastic and the pin, and the electrode wire will break cleanly at this point leaving no tail that could create cross-talk.
  - In case the electrode wire does not break cleanly, use scissors to cut extra wire.
18. Measure and trim the ends of the wires at the end of each cannula (Fig. 3*J*).
  - Cut at *z* mm (from formula above) past the end of each cannula.
  - The total length of the cannula and wire from the bottom of the bottom plastic piece can also be measured to be sure it is equal to the bregma depth specified in the design.
19. For the best chance of success, ensure each electrode meets all the following steps.
  - All connections between pins and wires are good and produce bubbles via the bubble test.
  - All ends of wires are trimmed.
    - The ends of wires around the pins are trimmed off to prevent cross-talk.
    - The ends of wires protruding from the cannula are trimmed to *z* mm past the end of the cannula.
  - All movable parts are glued in place as follows:
    - both plastic pieces to the dowel,
    - cannula to the bottom plastic piece,
    - pins to the top plastic piece,
    - wires where they enter the cannula.
  - The end of the ground and reference wire(s) are stripped.
  - All pins are clear, and a male end of a Mill-Max connector can be plugged in.
  - All cannulas and wires are as straight as they possibly can be.
  - The top plastic piece is well aligned with the bottom plastic piece.
  - All cannulas and wires are the proper lengths to hit each targeted structure.
20. For sterilization with ultraviolet light using a UV sterilizer, the steps are the following:
  - Place empty weigh boats in the UV sterilizer about 30 min prior to sterilizing the CMEA, and turn on the sterilizer.
  - Place the completed multielectrode array on one side in the weigh boat while the UV light is on.
  - With sterile gloves, turn the CMEA onto the other side after 30 min.
  - The CMEA will be ready to implant after another 30 min. Depending on the size of the UV sterilizer, multiple CMEAs can be sterilized simultaneously.

- The CMEA can also be sterilized via alternative methods such as ethylene oxide gas sterilization.

### Stereotaxic surgery

**Figure 4.**
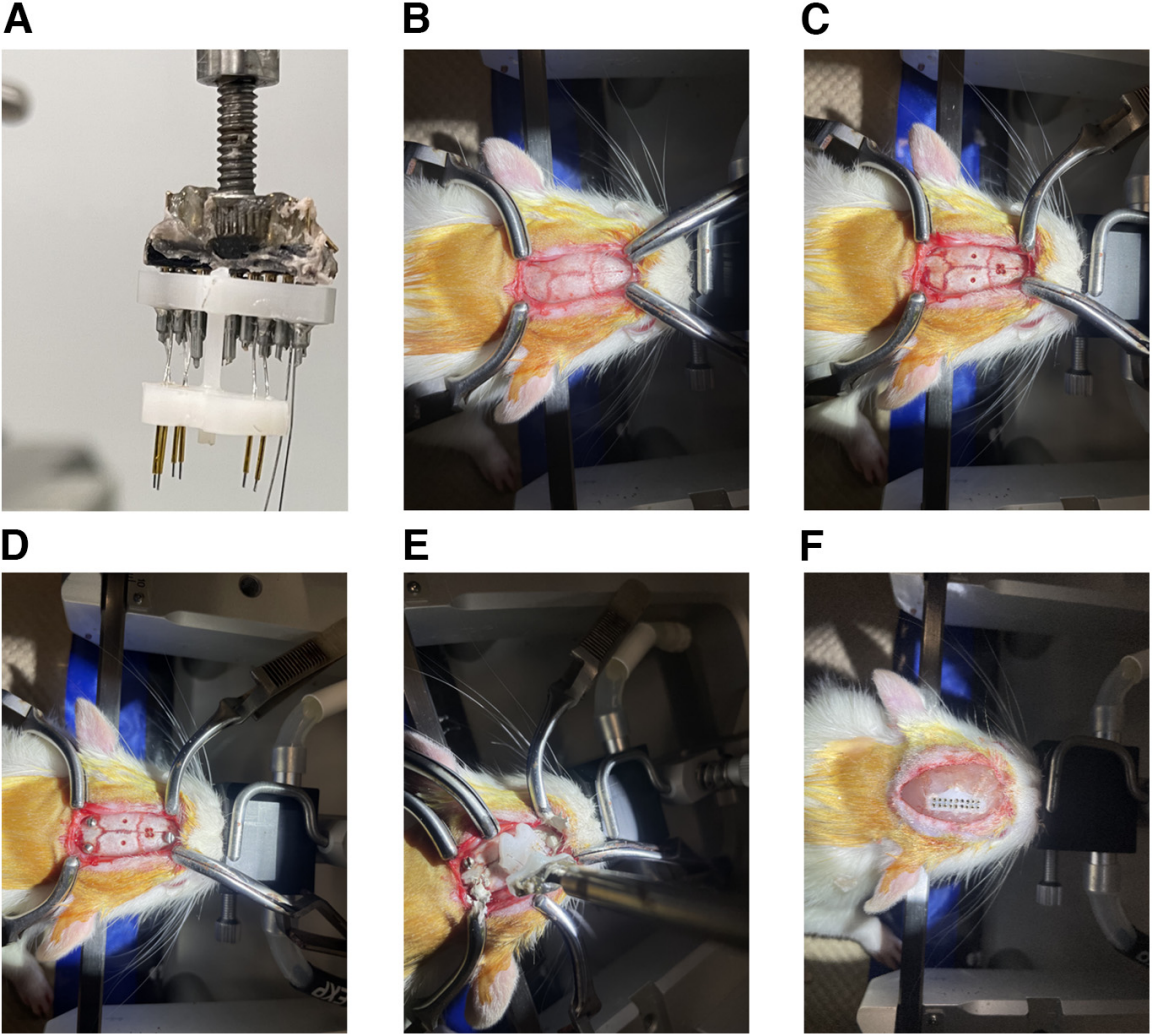
Stereotaxic surgery to implant multielectrode array. ***A***, Multielectrode array connected to the arm of the stereotaxic frame. ***B***, Cleaned skull showing lambda and bregma. ***C***, Holes drilled in the skull of the rat corresponding to coordinates of targeted brain regions. ***D***, Screws (ground, reference, and anchor) secured into the skull. ***E***, Multielectrode array inserted into the predrilled holes in the skull of the rat. ***F***, Dental cement was applied to the skull to secure the implanted multielectrode array on the head of the rat.

The unipolar CMEA was constructed to target different brain regions with coordinates obtained from the Paxinos rat brain atlas (Paxinos and Watson, 2013). To allow electrode implantation, the custom-built multielectrode array was first attached to one arm of a stereotaxic frame as shown in Figure 4*A*. An incision was made to expose the skull, and the skull was cleaned and dried (Fig. 4*B*); the dryness of the skull ensures patency and longevity of the implant. A stereotaxic arm-mounted drill was used to make holes (0.9 mm in diameter, totaling number of electrodes) in the skull (Fig. 4*C*) using stereotaxic coordinates (matching coordinates of the bottom plate) with bregma as a reference. This was done to doubly ensure that the electrodes will align accurately with the cannula holes in the bottom plastic plate. Afterward, a reference screw was secured into the skull behind lambda, and a ground screw was secured anterior to bregma (Fig. 4*D*). Anchor screws were also secured into the skull to help stabilize the dental cement (Fig. 4*D*). Subsequently, the multielectrode array was gently lowered down to 1 mm above the skull (Fig. 4*D*), and the electrodes were aligned to the corresponding drilled holes in the skull relatively easily. The reference and ground wires were then tied to the reference screw and the ground screw, and silver paint was applied on top of the screws to ensure connectivity (Fig. 4*E*). Glue was then applied on the skull surface before lowering the multielectrode array completely onto the skull surface. Dental cement was then applied to the skull and around the multielectrode array to provide stability and prevent any further movement (Fig. 4*F*).

**Figure 5.**
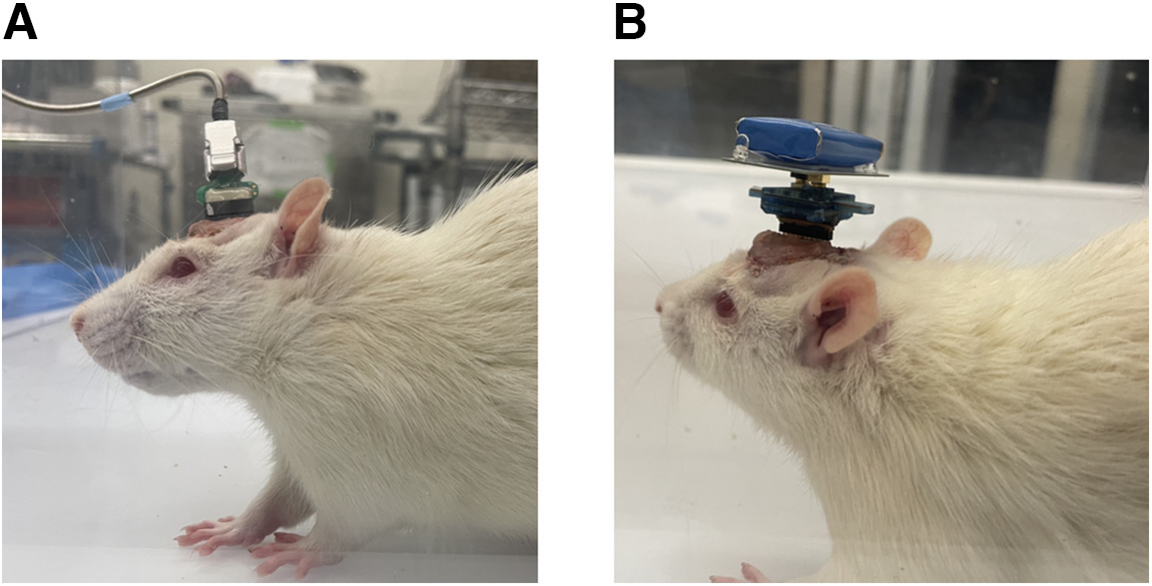
***A***, ***B***, Rat connected to the wired head stage for the Tucker Davies Technology RZ10 system (***A***) and wireless head stage for the W2100 system (***B***).

### Acquisition of local field potential data

Local field potential data were recorded using either the wired RZ10 Tucker Davis Technology (TDT) system or the wireless MultiChannel Systems W2100.

1. Interface the CMEA (16 channels) with the RZ10 Tucker Davies Technology (TDT) system as follows:
  - Using a wire cutter, cut a strip of the male/male ZIF-Clip to Mill-Max connector with nine pairs of gold pins (18 pins, including one pin for reference and one for ground).
  - Insert the pins from this precut Mill-Max connector into the sockets in the ZCA-Mil16 head stage adapter.
  - Connect the precut Mill-Max connector attached to the ZCA-Mil16 head stage adapter to the animal by inserting the protruding pins into the sockets in the CMEA on the head of the animal.
  - Attach the ZIF-Clip analog head stage to the ZCA-MIL16 head stage adapter and ensure that the ZIF-Clip is tightly secured on the head stage adapter (Fig. 5*A*).
2. Interface the CMEA (10 channels) with the MultiChannel Systems as follows:
  - Using a wire cutter, cut a strip of the male/male ZIF-Clip to Mill-Max connector with 10 gold pins (including one pin for ground).
  - Insert the pins from this precut Mill-Max connector into the sockets in the wireless W2100-HS116 head stage.
  - Connect the precut Mill-Max connector attached to the wireless head stage to the implanted multielectrode array (Fig. 5*B*).
  - The signal is collected using a wireless W2100 system (MultiChannel Systems).

**Figure 6.**
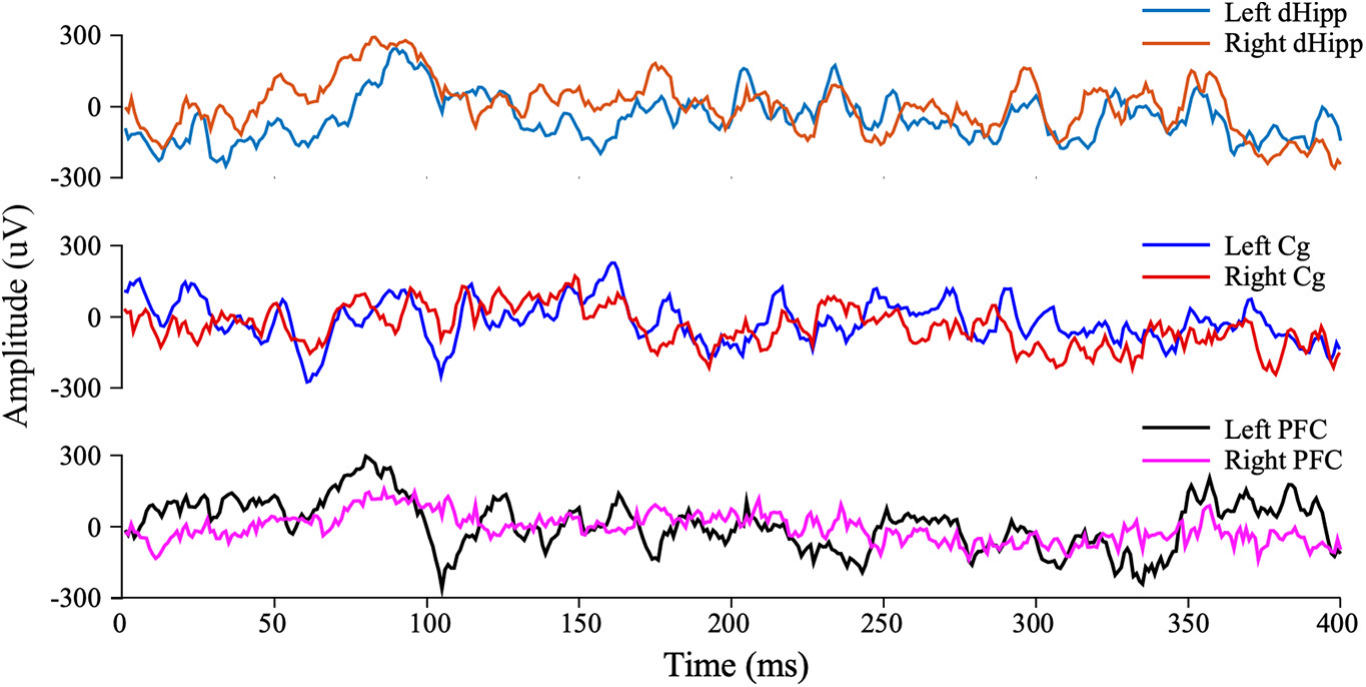
Representative traces of local field potential recordings from the left and right dorsal hippocampus, cingulate cortex, and prefrontal cortex.

LFP data collected can be analyzed using the Chronux software package for MATLAB (MathWorks). Example traces of acquired LFP data are shown in Figure 6.

## Discussion

Advances in electrophysiological recording have been crucial in understanding brain systems communication (Fielder et al., 2020; Quansah Amissah et al., 2021; Albeely et al., 2022). However, there are several challenges associated with collecting stable, long-term neuronal recordings from freely moving animals. These challenges include high cost, poor adaptability, and complexity of fabrication of both commercially available and custom-built recording electrodes. Here, a simple, lightweight, and affordable CMEA that can be used to record LFP activity from, and perform electrical stimulation of, multiple brain regions simultaneously in freely moving rats was described.

With all the required components available, it takes ∼1 h to build one probe for someone with no electrode-building experience. This time reduces significantly after gaining experience with the assembly. Moreover, the CMEA costs less than $16 Canadian for a 16-channel electrode, which is significantly cheaper than the cost of Michigan, Utah, and Neuropixels electrodes (Francoeur et al., 2021). These properties make the proposed CMEA attractive as a tool for recording LFP activity.

In this study, LFPs were recorded from three bilateral brain regions simultaneously. However, the plate was designed to enable the acquisition of LFP data from eight bilateral regions, regardless of their dorsoventral, anteroposterior, and mediolateral coordinates, using a 16-channel electrode. This makes it ideal for use in studies to explore neural activity in several brain regions simultaneously, and the interplay between these regions (e.g., coherence and cross-frequency coupling). This CMEA can also be used to record single-unit and multiunit neural activities if the 50 µm stainless steel metal wires being used as electrodes are replaced with bundles of four 12.5 µm (or less) metal electrodes. In this setting, these bundles would act as tetrodes but would not be drivable.

In general, how long an implanted electrode remains attached to the skull depends on a number of factors associated with the stereotaxic surgery performed to implant it, including attention to sterility during the surgery and how secure the implanted electrode was on the skull (Gardiner and Toth, 1999). Lack of attention to the sterility of the surgical tools and equipment could lead to infections, which will ultimately lead to the implant loosening and becoming dislodged. Additionally, not allowing the skull surface to dry properly before bone cement application could lead to inadequate attachment of the implant to the skull and cause the implant to be dislodged. Finally, inserting too few skull screws could also reduce the longevity of the implant. With proper routine cleaning of the skin around the implant, the CMEA implants have lasted for longer than 6 months following implantation with recordings of up to 1 h per session, with little to no effect on the LFP signal quality; however, the maximum length for continuous recordings has not been explored because of data storage limitations and to avoid potential duress to the animals.

One important feature of neural probes that could significantly affect the outcome of studies involving animal models is their weight. Probes must be lightweight to allow the animals to behave normally without any impairments and to obtain good-quality data for analysis and interpretation. The proposed CMEA is <1 g in weight (specifically, 735 mg for an 8-channel electrode for rats); thus, it is unlikely to affect the behavior of the animals following implantation. Animals have performed several tasks successfully without showing any signs of impairments in their behavior following implantation of the proposed CMEA (Albeely et al., 2022; Henricks et al., 2022; Albeely et al., 2023). Moreover, the compact shape of the CMEA makes it appropriate for recording neural activity during several types of behavioral experiments. The top and bottom plastic pieces can also be fabricated using a 3D printer instead of a CNC machine; however, because of the level of precision required, the pieces would have to printed using a high-resolution 3D printer such as a microSLA 3D printer, which is expensive and may not be accessible to all researchers. Therefore, CNC fabrication may be the cheapest and the most feasible option. The CMEA can also be scaled up or down further and adapted for experiments involving mice or nonhuman primates, making it adaptable for different species and experimental settings. This can be facilitated using different types of wire for recording (e.g., nichrome), as well as modifying the number of wires per cannula, which are also dependent on the thickness of the wire and gauge of the cannula. Furthermore, when considering this design for brain-stimulation-based studies, it is important to maximize the surface area to minimize charge density to prevent tissue damage (Kuncel and Grill, 2004). This requires larger-diameter wire, which can be further improved by deinsulating the bottom 1 mm of the stimulating wire.

Despite the advantages the proposed CMEA has over other commercially available ones, there are some limitations that are worth noting. First, because of the design of the top and bottom plastic plates, the number of sites that can be recorded simultaneously is limited. Unlike other commercially available electrodes like the Michigan and Utah electrodes, which can record from several hundred sites simultaneously, the proposed CMEA can record from a maximum of 16 sites at a time. Another limitation is that the proposed CMEA, once implanted in the brain, is fixed. It can therefore not be used in studies where multiple layers of specific brain regions are to be studied.

The proposed multielectrode array is cheap, easy, and fast to build and is mainly suited for LFP activity recording in awake, freely behaving animal models. Because of these advantages, they could be used in studies to investigate LFP and single-unit and multiunit activity in 16 brain regions simultaneously, and even those that require brain-stimulation-based approaches. This approach also allows electrophysiological studies to be performed in resource-restricted environments, making neuroscience accessible for the broadest possible range of institutions, especially in combination with other open-source tools (e.g., Open Ephys, OpenVape; Siegle et al., 2017; Frie et al., 2020).

## Synthesis

Reviewing Editor: Mark Laubach, American University Decisions are customarily a result of the Reviewing Editor and the peer reviewers coming together and discussing their recommendations until a consensus is reached. When revisions are invited, a fact-based synthesis statement explaining their decision and outlining what is needed to prepare a revision will be listed below. The following reviewer(s) agreed to reveal their identity: Matthew Gardner, Samantha White.

Please revise your manuscript to address the points raised below by two expert reviewers. Thank you for sending your work to eNeuro!

Reviewer 1

In this manuscript the authors introduce an open source method for recording local field potentials (LFPs) in vivo from multiple brain regions of rats using a cost-effective and simplified design. The design consists of single wires (stainless steel) which are used to perform unipolar recordings at each of the sites. In order to precisely target the multiple sites, the electrodes are attached to a CNC’d Delrin mold with predefined stereotaxic locations. The authors provide extremely detailed instructions on how to construct the customizable multielectrode array (CMEA), a list of the parts with pricing, and several pictures detailing the parts and steps of the assembly process. The authors further show how to make and employ adaptors for the mill-max connectors to headstages from the RZ10 TDT system (Tucker Davis Technologies) and the W2100 MultiChannel System (vendor?). Finally, the authors show examples of what appear to be high quality LFP (minimal noise) recordings indicating the viability of this system.

Overall I found the paper straightforward, easy to follow, and clearly effective in acquiring unipolar LFP recordings. The ability to record LFPs from a substantial number (4-16) of sites using an affordable and easy-to-build design is unquestionably a substantial benefit for neuroscientists not only in the field of in vivo electrophysiology, but also to behavioral neuroscience more generally, as it potentially makes these types of recordings much more approachable both from economic and ease-of-use considerations.

I have just a few questions for the authors:

1. I personally believe that LFP recordings are underutilized in the field, and provide a different, yet equally as important, measure of aggregate activity as compared to the increasingly popular method of fiber photometry. I suggest including a few sentences on the benefits of using LFPs to putatively assess communication between brain regions, such as field-field coherence, as this provides a measurement of network-level activity that not many other techniques can provide. Perhaps even comparing multi-site LFPs as an alternative assessment of activity to photometry would be helpful. This is just a recommendation to the authors.

2. Can the authors provide a bit more detail on some of the parts? I noticed that the authors included part numbers but left out vendor/manufacturer information in the parts list. Perhaps this was intentional, but it would be helpful to know exactly the parts that the authors used. Furthermore, a few other parts were not included in the parts table, such as the male/male zif-clip to mill-max adaptor.

3. I’m guessing that placement of the electrode isn’t always perfect when trying to match up the set of electrodes to the respective craniotomies. Can the authors provide information on how large the craniotomies need to be in general to get successful alignment, or how to handle this case when troubleshooting? Perhaps it doesn’t happen often, if not, perhaps just a statement that alignment tends to be fine.

4. This may be a simple oversight on my part, but I did not see any information for accessing the CAD files. Can the authors add information about where to access this information? If this was removed temporarily for the purpose of anonymous review, I apologize for mentioning this!

5. How is the mold aligned to bregma? This actually relates to question 3, and my guess is that alignment to bregma relies on alignment of the electrodes with holes.

6. Again maybe I missed a statement about this in the text, but can the authors make a qualitative statement about how long they’ve had the recordings be viable? I’m guessing that based on the design, the recordings should be robust for quite a long time, but a sense of what’s possible for recording time might be helpful.

Reviewer 2

Summary and Impressions: This manuscript outlines a detailed protocol for designing and building affordable microwire electrode arrays, useful for local field potential recordings. The step by step instructions make the process of building these electrodes seemingly simple, and the authors also provide an incredible amount of information related to the surgical implantation and recording process with their tool. The design of the electrodes itself is also well thought out, and seems straightforward to implant (which is not always the case with EEG/LFP systems in rodents). The data they use to demonstrate the feasibility of these electrodes is convincing. Overall, this manuscript is a stand-out example of a simple set of instructions for an otherwise complicated procedure, which helps to increase the accessibility of LFP recording methods to researchers interested in the topic. I have only one minor suggestion and two questions related to the accessibility of the project.

1) The included schematic of the electrode array is very useful, especially for demonstrating the diversity of areas that can be simultaneously recorded from a single implant. The description of the steps necessary to design the file are straightforward as well, and the text mentions that two samples are included which can be modified for brain regions of interest, however, it’s not clear how those files will be available. (If they will be available as supplementary materials, they were not attached to the review; a GitHub repository to host the files may also be useful).

2) The plastic pieces of the electrode implant are cut from Delrin with a CNC machine. What thickness of Delrin should be purchased to create these electrode plastic pieces? The authors should include this detail in the manuscript.

3) Would it be feasible to create the plastic piece with other common manufacturing methods such as 3D printing, or 3D resin printing? Why or why wouldn’t these alternatives be suitable to try? The authors might consider adding a statement about alternatives for manufacturing these plastic pieces, if they feel it would be reasonable to do so. This might increase accessibility of the design to laboratories without access to a CNC machine.

## Author Response

Responses to Reviewers:

Thank you to the reviewers for their continued insight and help with our manuscript. We have addressed the comments below.

Reviewer 1

In this manuscript the authors introduce an open-source method for recording local field potentials (LFPs) in vivo from multiple brain regions of rats using a cost-effective and simplified design. The design consists of single wires (stainless steel) which are used to perform unipolar recordings at each of the sites. In order to precisely target the multiple sites, the electrodes are attached to a CNC’d Delrin mold with predefined stereotaxic locations. The authors provide extremely detailed instructions on how to construct the customizable multielectrode array (CMEA), a list of the parts with pricing, and several pictures detailing the parts and steps of the assembly process. The authors further show how to make and employ adaptors for the mill-max connectors to headstages from the RZ10 TDT system (Tucker Davis Technologies) and the W2100 MultiChannel System (vendor?). Finally, the authors show examples of what appear to be high quality LFP (minimal noise) recordings indicating the viability of this system.

Overall I found the paper straightforward, easy to follow, and clearly effective in acquiring unipolar LFP recordings. The ability to record LFPs from a substantial number (4-16) of sites using an affordable and easy-to-build design is unquestionably a substantial benefit for neuroscientists not only in the field of in vivo electrophysiology, but also to behavioral neuroscience more generally, as it potentially makes these types of recordings much more approachable both from economic and ease-of-use considerations.

I have just a few questions for the authors:

1. I personally believe that LFP recordings are underutilized in the field, and provide a different, yet equally as important, measure of aggregate activity as compared to the increasingly popular method of fiber photometry. I suggest including a few sentences on the benefits of using LFPs to putatively assess communication between brain regions, such as field-field coherence, as this provides a measurement of network-level activity that not many other techniques can provide. Perhaps even comparing multi-site LFPs as an alternative assessment of activity to photometry would be helpful. This is just a recommendation to the authors.

We agree! We have now added sentences to the introduction section on lines 68 to 73 as follows: “One example of an invasive in vivo technique is fiber photometry, which relies on the expression of genetically encoded calcium indicators (GECI), that provides insight into neuronal activity by measuring changes in calcium levels (Legaria et al., 2022). The technique offers complementary advantages over EEG, in that it enables cell-type specific targeting and monitoring of neuronal activity and has better spatial resolution compared to EEG. Other invasive in vivo techniques involve the implantation of metal electrodes, which have low high-frequency impedance, high signal-to-noise ratio, and good mechanical properties (Im and Seo, 2016), in the brain to collect APs from neurons (O’Keefe and Dostrovsky, 1971; Ranck Jr, 1973).”

We also added the following sentences to the introduction section on lines 87 to 94: “While EEG, calcium, and LFP activity represent neural population activity, LFPs have a better spatial and temporal resolution, are stable and robust across changes in the electrode sensitivity (Im and Seo, 2016; Zhang et al., 2022), and are less susceptible to electrode micromotion effects (Martini et al., 2020). Moreover, LFPs can be used to evaluate field-field coherence, a measure of correlation between the activity of two brain regions in a specific frequency band (Fries, 2005). This can provide insights into the functional connectivity between different brain regions and how this changes over time (Aoi et al., 2015; Schneider et al., 2021). Some benefits of LFP recordings over photometry include the ease of not requiring viral injections and delays related to expression, as well as the potential to target a much greater number of sites.”

2. Can the authors provide a bit more detail on some of the parts? I noticed that the authors included part numbers but left out vendor/manufacturer information in the parts list. Perhaps this was intentional, but it would be helpful to know exactly the parts that the authors used. Furthermore, a few other parts were not included in the parts table, such as the male/male zif-clip to mill-max adaptor.

We have updated Table 1 with the vendor information and details for the male-male zero-insertion-force (ZIF) clip to mill-max adaptor.

3. I’m guessing that placement of the electrode isn’t always perfect when trying to match up the set of electrodes to the respective craniotomies. Can the authors provide information on how large the craniotomies need to be in general to get successful alignment, or how to handle this case when troubleshooting? Perhaps it doesn’t happen often, if not, perhaps just a statement that alignment tends to be fine.

First of all, we would like to clarify that we do not make craniotomies, but instead individual holes (0.9 mm in diameter) are drilled into the skull to allow for the passing of the cannulas and single wires. We have added the diameter to the text on lines 301 to 303 as follows: “A drill was used to label the targeted brain regions and make holes (0.9 mm in diameter) in the skull (Fig. 4C) using bregma as a reference.”

We also added a sentence on the ease of alignment of electrodes with the drilled holes on lines 307 to 309 as follows: “Subsequently, the multielectrode array was gently lowered down to 1 mm above the skull (Fig. 4D) and the electrodes were aligned to the corresponding drilled holes in the skull relatively easily.”

4. This may be a simple oversight on my part, but I did not see any information for accessing the CAD files. Can the authors add information about where to access this information? If this was removed temporarily for the purpose of anonymous review, I apologize for mentioning this!

We have provided information on where the files can be accessed on lines 141 to 145 as follows: “Based on the schematic (Fig. 2), which contains the brain regions of interest and their coordinates (anterior-posterior and medial-lateral), the models for the top and bottom plastic pieces were created in a computer-aided design (CAD) software (two sample wireframe IGES files have been included (see Doi 10.17605/OSF.IO/ZXF5T) that can be modified for the brain regions of interest).”

5. How is the mold aligned to bregma? This actually relates to question 3, and my guess is that alignment to bregma relies on alignment of the electrodes with holes.

We have added a sentence to the text on lines 301 to 304 to address this comment as follows: “A A stereotaxic arm-mounted drill was used to make holes (0.9 mm in diameter; totaling number of electrodes) in the skull (Fig. 4C) using stereotaxic coordinates (matching coordinates of the bottom plate) with bregma as a reference. This was done to doubly ensure that the electrodes will align accurately with the cannula holes in the bottom plastic plate.”

6. Again maybe I missed a statement about this in the text, but can the authors make a qualitative statement about how long they’ve had the recordings be viable? I’m guessing that based on the design, the recordings should be robust for quite a long time, but a sense of what’s possible for recording time might be helpful.

We have recorded for up to one hour per session and, as mentioned in the manuscript, recordings have been performed in our animals for up to six months using our CMEA.

We have now added a sentence to the text on lines 394 to 398 to this effect: “With proper routine cleaning of the skin around the implant, the CMEA implants have lasted for longer than 6 months following implantation with recordings of up to one hour per session, with little to no effect on the LFP signal quality; however, the maximum length for continuous recordings have not been explored due to data storage limitations and to avoid potential duress to the animals.”

Reviewer 2

Summary and Impressions: This manuscript outlines a detailed protocol for designing and building affordable microwire electrode arrays, useful for local field potential recordings. The step by step instructions make the process of building these electrodes seemingly simple, and the authors also provide an incredible amount of information related to the surgical implantation and recording process with their tool. The design of the electrodes itself is also well thought out, and seems straightforward to implant (which is not always the case with EEG/LFP systems in rodents). The data they use to demonstrate the feasibility of these electrodes is convincing. Overall, this manuscript is a stand-out example of a simple set of instructions for an otherwise complicated procedure, which helps to increase the accessibility of LFP recording methods to researchers interested in the topic. I have only one minor suggestion and two questions related to the accessibility of the project.

1. The included schematic of the electrode array is very useful, especially for demonstrating the diversity of areas that can be simultaneously recorded from a single implant. The description of the steps necessary to design the file are straightforward as well, and the text mentions that two samples are included which can be modified for brain regions of interest, however, it’s not clear how those files will be available. (If they will be available as supplementary materials, they were not attached to the review; a GitHub repository to host the files may also be useful).

We have provided information on where the files can be accessed on lines 141 to 145 as follows: “Based on the schematic (Fig. 2), which contains the brain regions of interest and their coordinates (anterior-posterior and medial-lateral), the models for the top and bottom plastic pieces were created in a computer-aided design (CAD) software (two sample wireframe IGES files have been included (see Doi 10.17605/OSF.IO/ZXF5T) that can be modified for the brain regions of interest).”

2. The plastic pieces of the electrode implant are cut from Delrin with a CNC machine. What thickness of Delrin should be purchased to create these electrode plastic pieces? The authors should include this detail in the manuscript.

The Delrin that was purchased for the fabrication of the top and bottom plastic pieces were 6 mm thick. We have modified the text on lines 146 to 151 to include the dimensions of the Delrin plates used as follows: “The model was then converted into G-CODE, using computer-aided manufacturing, and uploaded into a computer numerically controlled (CNC) machine to carve out the templates for the top and bottom plastic pieces from the Delrin (Dupont Canada, Kingston) plates (150 x 150 x 6 mm) according to the dimensions specified in the CAD file.”

3. Would it be feasible to create the plastic piece with other common manufacturing methods such as 3D printing, or 3D resin printing? Why or why wouldn’t these alternatives be suitable to try? The authors might consider adding a statement about alternatives for manufacturing these plastic pieces, if they feel it would be reasonable to do so. This might increase accessibility of the design to laboratories without access to a CNC machine.

Due to the level of precision required to reach the brain regions of interest, the electrode templates would have to printed using a high resolution 3D printer, which is expensive and may not be accessible to some researchers. We have added a sentence to the discussion section on lines 408 to 412 as follows: “The top and bottom plastic pieces can also be fabricated using a 3D printer instead of a CNC machine; however, due to the level of precision required, the pieces would have to printed using a high-resolution 3D printer such as a microSLA 3D printer, which is expensive and may not be accessible to all researchers. Therefore, CNC fabrication may be the cheapest and the most feasible option.”
