# Supplementary material for: A Simple, Lightweight, and Low-Cost Customizable Multielectrode Array for Local Field Potential Recordings
Source: eNeuro. 2023 Aug 28;10(8):ENEURO.0212-23.2023. doi: 10.1523/ENEURO.0212-23.2023 (PMC10467017; doi:10.1523/ENEURO.0212-23.2023)
Supplement: Table 1-1 — Alternative supplier list. Download Table 1-1, DOCX. [file enu-eN-OTM-0212-23-s01.docx]

| **Item** | **Product Number** | **Vendors** | **Quantity** |  | **Cost per CMEA** |
| --- | --- | --- | --- | --- | --- |
| [Delrin Plastic Pieces](https://www.globalindustrial.ca/p/white-acetal-plastic-bar-332-thick-x-34-wide-x-12-long?infoParam.campaignId=T9F&utm_source=google&utm_medium=cpc&utm_campaign=Catch_All_-_Weekday_-_Dropship&utm_term=&utm_content=222116844165&gclid=EAIaIQobChMIsIz9kcGd_wIVmczjBx2oTQSDEAYYCCABEgKl8_D_BwE) | T9FB2364713 | Global Industrial | 1 |  | 2.05 |
| [Plastic dowel](https://www.mcmaster.com/products/acetal-dowel-pins/) | 97155A115 | McMaster Carr | 1 |  | 0.11 |
| [Polyimide Tubing](https://www.a-msystems.com/p-219-polyimide-tubing.aspx) | 822000 | A-M Systems | 1 (33 mm) |  | 1.02 |
| [PFA-coated Stainless Steel Wire](https://componentsupplycompany.com/stainlesssteelwire.php?gad=1&gclid=EAIaIQobChMIuoa35fGd_wIVhhKtBh3MZgq7EAAYASAAEgLDuPD_BwE) | GWX-0050 | CS Component Supply | 1 (18 in) |  | 3.5 |
| [Mill-Max Connector](https://www.digikey.ca/en/products/detail/würth-elektronik/62202821821/15672173?utm_adgroup=Connectors%2C%20Interconnects&utm_source=google&utm_medium=cpc&utm_campaign=Shopping_Supplier_Würth%20Elektronik_8082_Co-op&utm_term=&productid=15672173&gclid=EAIaIQobChMImKKN1Oyd_wIVOhqtBh1ZUwRpEAYYASABEgKObvD_BwE) | 62202821821 | DigiKey | 1 (2 x 9 pins) |  | 3.81 |
| [Super Glue](https://www.uline.ca/Product/Detail/S-17189/Adhesives-Glue-Epoxy/Loctite-Instant-Adhesive-444-Tak-Pak) | S-17189 | ULINE | 1 (100 μL) |  | 0.175 |
|  |  |  |  |  |  |
| **Total cost** |  |  |  |  | **CAD$ 10.507** |
